# microRNA 126 Inhibits the Transition of Endothelial Progenitor Cells to Mesenchymal Cells via the PIK3R2-PI3K/Akt Signalling Pathway

**DOI:** 10.1371/journal.pone.0083294

**Published:** 2013-12-13

**Authors:** Junfeng Zhang, Zongqi Zhang, David Y. Zhang, Jianbing Zhu, Tiantian Zhang, Changqian Wang

**Affiliations:** 1 Department of Cardiology, Ninth People's Hospital, Shanghai Jiao Tong University School of Medicine, Shanghai, China; 2 Department of Cardiology, Third People's Hospital, Shanghai Jiao Tong University School of Medicine, Shanghai, China; 3 Section of Cardiology, Department of Medicine, The University of Chicago Pritzker School of Medicine, Chicago, Illinois, United States of America; University of Barcelona, Spain

## Abstract

**Aims:**

Endothelial progenitor cells (EPCs) are capable of proliferating and differentiating into mature endothelial cells, and they have been considered as potential candidates for coronary heart disease therapy. However, the transition of EPCs to mesenchymal cells is not fully understood. This study aimed to explore the role of microRNA 126 (miR-126) in the endothelial-to-mesenchymal transition (EndMT) induced by transforming growth factor beta 1 (TGFβ1).

**Methods and Results:**

EndMT of rat bone marrow-derived EPCs was induced by TGFβ1 (5 ng/mL) for 7 days. miR-126 expression was depressed in the process of EPC EndMT. The luciferase reporter assay showed that the PI3K regulatory subunit p85 beta (PIK3R2) was a direct target of miR-126 in EPCs. Overexpression of miR-126 by a lentiviral vector (lenti-miR-126) was found to downregulate the mRNA expression of mesenchymal cell markers (α-SMA, sm22-a, and myocardin) and to maintain the mRNA expression of progenitor cell markers (CD34, CD133). In the cellular process of EndMT, there was an increase in the protein expression of PIK3R2 and the nuclear transcription factors FoxO3 and Smad4; PI3K and phosphor-Akt expression decreased, a change that was reversed markedly by overexpression of miR-126. Furthermore, knockdown of PIK3R2 gene expression level showed reversed morphological changes of the EPCs treated with TGFβ1, thereby giving the evidence that PIK3R2 is the target gene of miR-126 during EndMT process.

**Conclusions:**

These results show that miR-126 targets *PIK3R2* to inhibit EPC EndMT and that this process involves regulation of the PI3K/Akt signalling pathway. miR-126 has the potential to be used as a biomarker for the early diagnosis of intimal hyperplasia in cardiovascular disease and can even be a therapeutic tool for treating cardiovascular diseases mediated by the EndMT process.

## Introduction

Coronary heart disease is a major cause of death and disability worldwide, and this disease is initiated by vascular endothelial injury. When the vascular endothelium is injured, circulating endothelial progenitor cells (EPCs), which are positive for both surface markers CD34 and KDR, are mobilized from the bone marrow (BM), migrate to the ischaemic tissue, differentiate to mature vascular endothelial cells, and then to repair the injured endothelium [1]. 

However, BM-derived EPCs also have the ability to transdifferentiate into a smooth muscle cell lineage positive for alpha smooth muscle actin (α-SMA), i.e., endothelial-to-mesenchymal transition (EndMT) [2], suggesting a contributive role for EPCs in intimal hyperplasia during the endothelial repair process. This role was supported by published results that EPCs promote an increase in the thickness of the intimal layer in the pulmonary arteries of patients with chronic obstructive pulmonary disease [3] and that EPCs stimulate late intimal hyperplasia in porcine arteriovenous expanded polytetraflouroethylene grafts [4]. Importantly, it has been demonstrated that BM-derived EPCs can migrate into the intimae of a balloon-injured carotid artery to augment intimal hyperplasia [5], which is secondary to the process of EndMT induced by the transforming growth factor beta 1 (TGF-β1) in the hypertrophic neointima [6]. However, the mechanisms underlying EPC involvement in intimal hyperplasia have not yet been fully elucidated.

microRNAs (miRNAs; miRs) are 21-23 nucleotides long, highly conserved, non-protein-coding RNAs; they mainly target the 3′-UTR of an mRNA to regulate gene expression at the post-transcriptional level by inhibiting the translation of a protein or by promoting mRNA degradation [7,8]. Microarray-based expression profiles in our previous study have identified several miRNAs in EPCs, such as microRNA 21 (miR-21), microRNA 27a (miR-27a), and microRNA 126 (miR-126) [9]. Furthermore, several studies have demonstrated that miR-21[10] and miR-127[11] are involved in mature endothelial cell EndMT induced by TGF-β1 and TGF-β2, respectively. 

One of the endothelial-specific microRNAs is miR-126 (miRBase accession no. MIMAT0000445). MicroRNA-126 is located within 7the intron of epidermal growth factor-like domain 7 (Egfl7) and is highly expressed in vascular endothelial cells [12]. miR-126* (miRBase accession no. MIMAT0000444), the partner to miR-126 that is derived from the same precursor transcript within the Egfl7 ghen, forms a miRNA pair with miR-126 to exert physiological functions. miR-126/miR-126* expression is downregulated in cancer cells by promoter methylation of their host gene Egfl7[13]. Studies have shown that miR-126 does not affect the differentiation of EPCs into mature endothelial cells [9], although it favours the proliferation, mobilization, and migration of EPCs [14,15]. In the animal model of mice and zebra fish, the loss-of-function study by deleting the targeted miR-126 gene leads to loss of vascular integrity during development and then result in defective angiogenesis[16][17] Additional study has demonstrated that miR-126 can positively regulate the response of ECs to vascular endothelial growth factor (VEGF), and improve angiogenesis in part by directly repressing negative regulators of the VEGF pathway, through mechanism involving the sprouty-related protein 1 (SPRED1) and phosphoinositol-3 kinase regulatory subunit 2 (PIK3R2)[18]. However, the role of miR-126 in regulating the transition of EPCs to mesenchymal cells has not been reported to date.

Recent studies have demonstrated that the gene coding for the phosphoinositide 3-kinase (PI3K) regulatory subunit p85 beta (*PIK3R2*) is one of the targets of miR-126 [19]. In vascular endothelial cells, miR-126 could negatively regulate the PI3K/Akt signalling pathway by targeting *PIK3R2* [16]. Furthermore, repression of phosphor-PI3K/Akt could inhibit the localization of the FoxO3/Smad4 transcription factor complex from the cytoplasm to the nucleus and then deactivate the Smad2/3-dependent signalling pathway [20], the activation of which has been proven responsible for the EndMT process of EPCs [5]. These results led us to hypothesize that miR-126 targets *PIK3R2* to facilitate PI3K/Akt activation and then to negatively modulate the Smad2/3-dependent signalling pathway to repress the EPC EndMT process.

In the present study, we assessed the effects of miR-126 on TGFβ1-induced EndMT of EPCs isolated from the bone marrow (BM). Additionally, we investigated the roles of PIK3R2 and the PI3K/Akt signalling pathway in the EndMT process of EPCs regulated by miR-126.

## Materials and Methods

The investigation conformed to the principles outlined in the U.S. National Institutes of Health guidelines for the use of animal tissues and was approved by the Ethics Committee of Experimental Research, Shanghai Jiao Tong University School of Medicine.

### Cells isolation, identification, and culture

BM-derived EPCs were isolated from male Sprague-Dawley rats (weight, 300–450 g), as described previously with minor modifications [5]. Briefly, mononuclear cells from the rat bone marrow were isolated with Ficoll-Isopaque Plus (Histopaque-1077; Sigma) density-gradient centrifugation. Isolated cells were resuspended in endothelial cell growth medium (EGM2; Lonza) composed of endothelial cell basal medium-2 (EBM-2) containing 20% FBS, and then cells were cultured on a fibronectin-coated dish at 37°C in a 5% CO_2_ incubator. Non-adherent cells were removed after 4–7 days by gentle washes with phosphate-buffered solution (PBS). The attached cells were used in the subsequent experiments. For identification, resuspended cells were incubated with PE-CD34 and FITC-KDR antibodies (Jackson IRL, Baltimore, MD), and CD34/KDR double-positive cells were identified as EPCs by using flow cytometry (FACScan^®^; BD Biosciences) and the CellQuest software (BD Biosciences).

### Induction of EndMT in EPCs

The cells were seeded on culture plates with Ham’s F-12 containing 5% FBS, and EPC EndMT was induced by TGF-β1 (5 ng/mL) for 7 days, as described previously [2]. 

In the experiment designed to explore the role of PIK3R2 in EndMT process, EPCs were treated with LY294002 (25 μM, Cell Signaling Technology, Beverly, MA; catalog no.9901) for one hour to block PI3K/Akt activation, or were transfected with PIK3R2siRNA according to the manufacturer’s protocol (Santa Cruz Biotechnology, Santa Cruz, CA; catalog no. 156022). Then the cells were used in subsequent experiments.

### Lentiviral Constructs, Packaging, and Transduction

We followed the protocol described previously from our lab[9]. The Expression plasmids for miR-126 was created using PCR amplification with rat genomic DNA as templates. The Primers are described in Table 1. The PCR product of miR-126 was cloned into PLKO.1-puro vector (Sigma-Aldrich). The construct of miR-126 was confirmed by sequencing. To produce lentivirus, the plasmid DNA was individually transfected into 293 T cells using psPAX2, a pMD2G packaging construct, and lipofectamine plus reagent (Invitrogen) according to the manufacturer’s protocol. After 6 h, the medium was refreshed, and viral supernatant was collected 48 h later. EPCs were seeded into 6-well plates (5×10^5^ cells per well) and cells were transfected with different lentiviral vectors at an MOI of 10. 48 h after infection, cells were selected and cloned by culture in the presence of puromycin (2 µg/mL) for 1 week. 

**Table 1:** PCR primers used for cDNA. Real-time PCR were carried out using the following primers:.

A	miR-126	F	5'-GCTGTCAGTTTGTCAAATAC-3'
		R	5'-GTGCAGGGTCCGAGGT-3'
B	miR-126*	F	5’-GGGCATTATTACTTTTGG-3’
		R	5’- TGCGTGTCGTGGAGTC-3’
C	U6	F	5'-CTCGCTTCGGCAGCACA-3'
		R	5'-AACGCTTCACGAATTTGCGT-3'
D	CD31	F	5'-AGTCAGTAAATGGGACTGCACCCA-3'
		R	5'-TCTCTGGTGGGCTTGTCTGTGAAT-3'
E	CD34	F	5'-AGACTCAGGGAAAGGCCAATGTGA-3'
		R	5'-GCCACCACATGTTGTCTTGCTGAA-3'
F	CD133	F	5'-AACGTGGTCCAGCCGAATG-3'
		R	5'-TCCCAGGATGGCGCAGATA-3'
G	KDR	F	5'-AGTGGCTAAGGGCATGGAGTTCTT-3'
		R	5'-GGGCCAAGCCAAAGTCACAGATTT-3'
H	α-SMA	F	5'-AATATTCTGTCTGGATCGGCGGCT-3'
		R	5'-GAAGCATTTGCGGTGGACAATGGA-3'
I	sm22-α	F	5'-AAGATATGGCAGCAGTGCAGAGGA-3'
		R	5'-CCATTTGAAGGCCAATGACGTGCT-3'
J	myocardin	F	5'-ACGGGATGGAGGTTTCTGTGACAA-3'
		R	5'-GGCACATTTCGAATGCATCACCGA-3'
K	VEGF	F	5'-TCACCAAAGCCAGCACATAGGAGA-3'
		R	5'-TTTCTCCGCTCTGAACAAGGCTCA-3'
L	Flt-1	F	5'-TAGATGTCCAAATAAGCCCGCCGA-3'
		R	5'-TCAATCCGCTGCCTGATAGATGCT-3'
M	eNOS	F	5'-TATTTGATGCTCGGGACTGCAGGA-3'
		R	5'-ACGAAGATTGCCTCGGTTTGTTGC-3'
N	iNOS	F	5'-TGATCTTGTGCTGGAGGTGACCAT-3'
		R	5'-TGTAGCGCTGTGTGTCACAGAAGT-3'
O	β-actin	F	5'-AGCACAGAGCCTCGCCTTTG-3'
		R	5'-ACATGCCGGAGCCGTTGT-3'
P	PIK3R2	F	5'-GCACCACGAGGAACGCACTT-3'
		R	5'-CGTCCACTACCACGGAGCAG-3'
Q	Egfl7	F	5’-CTCTCCTACCCACAGCAATATG-3’
		R	5’-CCTCTCCTGTACTGCATTCATC-3’

### Quantitative real-time PCR analysis

Total RNA was extracted using Trizol reagent (Invitrogen) according to the manufacturer’s instructions. Extracted RNA was further purified and reverse-transcribed into cDNA by using the TaqMan MicroRNA Reverse Transcription Kit (Applied Biosystems, USA) along with specific primers (Table 1). Quantitative real time-PCR (qRT-PCR) was performed using SYBR Green PCR master mix (Applied Biosystems) on an ABI 7500HT System. U6 snRNA (for miR-126) and β-actin (for mRNAs) were used as endogenous controls. The primer sequences used are listed in Table 1. All samples were normalized to internal controls, and the relative expression level was calculated through the 2^−ΔΔCt^ analysis method. Experiments were performed in triplicate samples. 

### Luciferase reporter assay

The luciferase-3′-UTR reporter plasmid containing a *PIK3R2* 3′-UTR carrying a putative or a mutant miR-126 binding site was constructed and cloned downstream of a cytomegalovirus (CMV) promoter-driven firefly luciferase cassette in a pCDNA3.0 vector. Luciferase assays were performed in a 24-well format. BM-derived EPCs cells were seeded into 24-well plates (1×10^5^ cells/well) by using lipofectamine 2000 according to the manufacturer’s protocol. Cells were then transfected with the *PIK3R2* 3′UTR wild type (wt) plasmid or the *PIK3R2* 3′UTR mutant (mt) plasmid in the presence of either miR-126 or miR-control. In addition, these cells were co-transfected with 0.1 µg of the *PIK3R2* 3′UTR firefly luciferase report vector and 0.02 µg pRL-TK Renilla vector (Promega) for normalization of transfection. After 48 h, cells were washed and lysed with passive lysis buffer. Then both firefly luciferase and renilla activity were determined using the dual-luciferase reporter assay system and a luminometer (Promega, WI)[21]. Protein content in each lysate was determined by Bio-Rad Protein assay (Bio-Rad). Luciferase activity was corrected for both protein content and renilla activity to account for cell density and transfection efficiency, respectively. 

### Immunofluorescence staining for αSMA

Cells were rinsed with PBS and then fixed with 4% paraformaldehyde containing 0.2% Triton X-100 for 15 minutes at room temperature. The cells were then blocked for 2 hours in PBS containing 5% normal goat serum and 2% bovine serum albumin. Monoclonal antibody (diluted 1:1000) against αSMA (Millipore Corporation, Billerica, MA; catalog no. CBL171) was incubated with the fixed and permeabilized cells for 2 hours, followed by 5 rinses with PBS. The cells then were incubated with Alexa594-conjugated rabbit anti-mouse antibody (diluted 1:500) for 1 hour. Cell nuclei were stained with 1 μg/mL DAPI for 5 minutes at room temperature. The fluorescence intensity was calculated using the Image J software (downloaded from http://rsbweb.nih.gov/ij/). The data have been represented as mean ± SD values for the intensity of pixels. 

### Western blot analysis

The proteins from cell lysates and nuclear extracts were prepared as described previously [22], separated with 10% SDS-PAGE, electrophoretically transferred to PVDF membranes, and probed with primary antibody followed by horseradish peroxidase-conjugated secondary antibodies. The primary antibodies for Western blot analysis were as follows: anti-PIK3R2 (Santa Cruz Biotechnology, Santa Cruz, CA; catalog no. sc-131324), anti-PI3K (Cell Signaling Technology, Beverly, MA; catalog no.4249S), anti-p-Akt (Cell Signaling Technology; catalog no. 9271S), anti-AKT (Cell Signaling Technology; catalog no.9272S), anti-α-SMA (Millipore Corporation, Billerica, MA; catalog no. CBL171), anti-CD31 (Santa Cruz Biotechnology; catalog no. sc-1506), anti-FoxO3 (Santa Cruz Biotechnology; catalog no. sc-9813), anti-Smad4 (Santa Cruz Biotechnology; catalog no. sc-7966), anti-β-actin (Cell Signaling Technology; catalog no.8456), and anti-lamin A (Abcam, Cambridge, UK; catalog no. ab26300). The membranes were developed with enhanced chemiluminescence reagent for visualization. 

As a method of quality control in our lab protocol, we routinely screened the purity of the nuclear protein by Western blot to check cytoplasm protein extract with anti-Lamin A antibody, and to check the nuclear protein extract with anti-β-actin antibody (unpublished data).

### Statistical analysis

The SPSS 13.0 software was used for statistical analysis. All data are expressed as the mean ± SD values of 3 independent experiments; each was performed in triplicate and analysed using ANOVA followed by the least significant difference (LSD) *t*-test for post-hoc comparison. *P* < 0.05 (*) and *P* < 0.01 (**) were considered statistically significant. 

## Results

### miR-126 expression decreases in EPC EndMT

**Figure 1:**
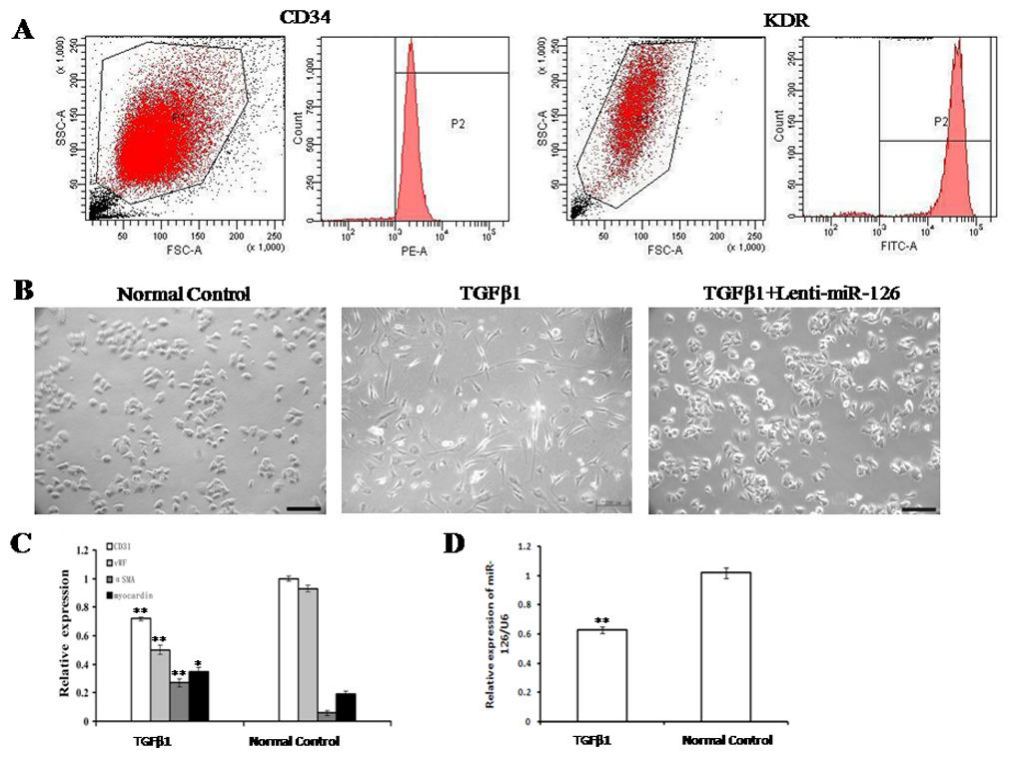
TGFβ1-induced EPCs underwent EndMT. (A) Flow cytometry analysis showed that 95% of cells were positive for both CD34 and *KDR*. (B) After the treatment with TGFβ1 (5 ng/mL) for 7 days, the morphology changed in EPCs (original magnification ×200, Bar=200 μm). (C) The mRNA expression levels of endothelial cell markers (CD31 and vWF) and mesenchymal cell markers (α-SMA and myocardin) in TGFβ1-induced EPCs were determined by using qRT-PCR. (D) After the treatment with TGFβ1 (5 ng/mL) for 7 days, miR-126 expression in EPCs was determined by using qRT-PCR. EPCs without any treatment were used as the normal control. Data are shown as mean ± S.D. (n = 3). **, *P* < 0.01 compared with the normal control.. The number of observations (n) represents the number of independent cell preparations.

As shown in Figure 1A, from the isolated EPCs, more than 95% were positive for both CD34 and KDR. After the treatment with TGFβ1 (5 ng/mL) for 7 days, EPCs underwent a morphological change from a cobblestone-like to a spindle-shaped appearance, while overexpression of miR-126 partially reversed the morphological changes (Figure 1B). After 7-day stimulation with TGFβ1 (5 ng/mL), EPCs showed decreased mRNA expression of the endothelial markers *CD31* and *vWF* and increased mRNA expression of the mesenchymal markers αSMA and myocardin (Figure 1C). In addition, miR-126 expression was simultaneously downregulated by the TGFβ1 (5 ng/mL) treatment for 7 days compared to the normal control (Figure 1D). miR-126* (miRBase accession no. MIMAT0000444), the partner to miR-126 that is derived from the same transcript within Egfl7 gene, had a similar expression pattern in EPCs when treated with TGFβ1 for 7 days course (Figure S3A). To investigate whether if miR-126/miR-126* host gene Egfl7 could also be involved the mechanism of EndMT in EPCs, quatitative real-time PCR did not show any difference of Egfl7 mRNA expression in the presence of TGFβ1 (. Figure S3B). Next, we demonstrated that overexpression of miR-126 had no effect of expression level of its host gene Egfl7 (. Figure S3B). 

**Figure 2:**
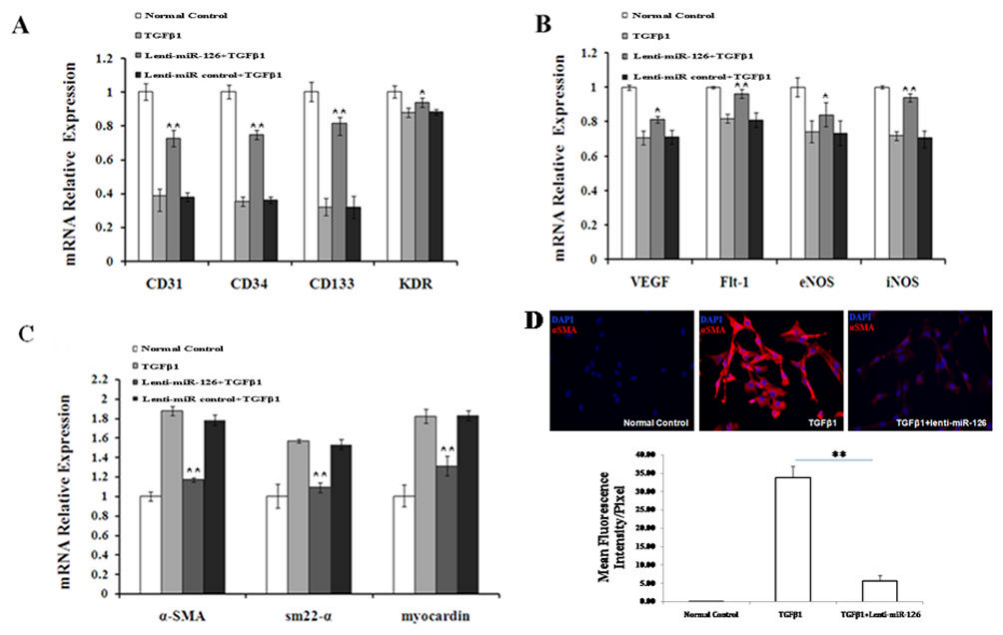
Overexpression of miR-126 inhibited the EndMT process of EPCs induced by TGFβ1. (A) The mRNA expression of progenitor cell markers (CD31, CD34, CD133, and *KDR*), (B) endothelial cell markers (VEGF, *Flt-1*, eNOS, and iNOS), and (C) mesenchymal cell markers (α-SMA, *sm22-α*, and myocardin) was determined by using qRT-PCR. (D) Protein expression of α-SMA was detected by immunofluorescence staining. EPCs induced by TGFβ1 were transfected with a miR-126-expressing lentiviral vector, an empty vector (lentivector), or neither, and EPCs without any treatment were used as the normal control. EPCs infected with an empty vector were used as the Lenti-miR control. Data shown are representative of 3 independent experiments. **, *P* < 0.01 compared with the Lenti-miR control.

Early EPCs express surface markers, such as CD133, KDR and CD34. However, late EPCs notably express CD34 and KDR, not CD133. In our study, we examined the expressions of the markers in EPCs (Figure 1A and Figure 2A). We found that all three markers of CD133, KDR and CD34 were markedly expressed in EPCs used in the present study, implying our research conclusion can be applied to early EPCs instead of late EPCs.

### Overexpression of miR-126 inhibits TGFβ1-induced EndMT of EPCs

To explore the role of miR-126 in TGFβ1-induced EndMT of EPCs, EPCs were transfected with a lentiviral vector (lenti-miR-126) to overexpress endogenous miR-126, and an empty control lentiviral vector was used as a control (marked as lenti-miR control). The infection efficiency of lenti-miR-126 was 87.56%, and endogenous miR-126 expression in lenti-miR126 transfected EPCs was 3.6-fold higher than that in the control (Figure S1). 

Real-time PCR was used to analyze the EndMT process of EPCs induced by TGFβ1. TGFβ1 treatment markedly decreased the mRNA expression of progenitor cell markers (*CD34*, *CD31*, *CD133*, and *KDR*) and endothelial cell markers (*VEGF*, *Flt-1*, *eNOS*, and *iNOS*), while overexpression of miR-126 increased the mRNA expression of the corresponding markers compared to the miR-126 control (Figure 2A & 2B). Analyses of mRNA levels for mesenchymal markers (α*-SMA*, *sm22-α*, and myocardin) showed that the expression levels of mesenchymal-derived markers were increased only by TGFβ1, while they were decreased in the presence of both miR-126 and TGFβ1 compared to the miR-126 control (Figure 2C). Immunofluorescence staining showed that the α-SMA protein was synthesized in the cytoplasm of EPCs treated with TGFβ1; however, the cytoplasmic levels of α-SMA were significantly lowered by miR-126 (Figure 2D). Overall, overexpression of miR-126 partially reversed the TGFβ1-induced EndMT process in EPCs. 

### PIK3R2 is a direct target of miR-126 in EPCs

**Figure 3:**
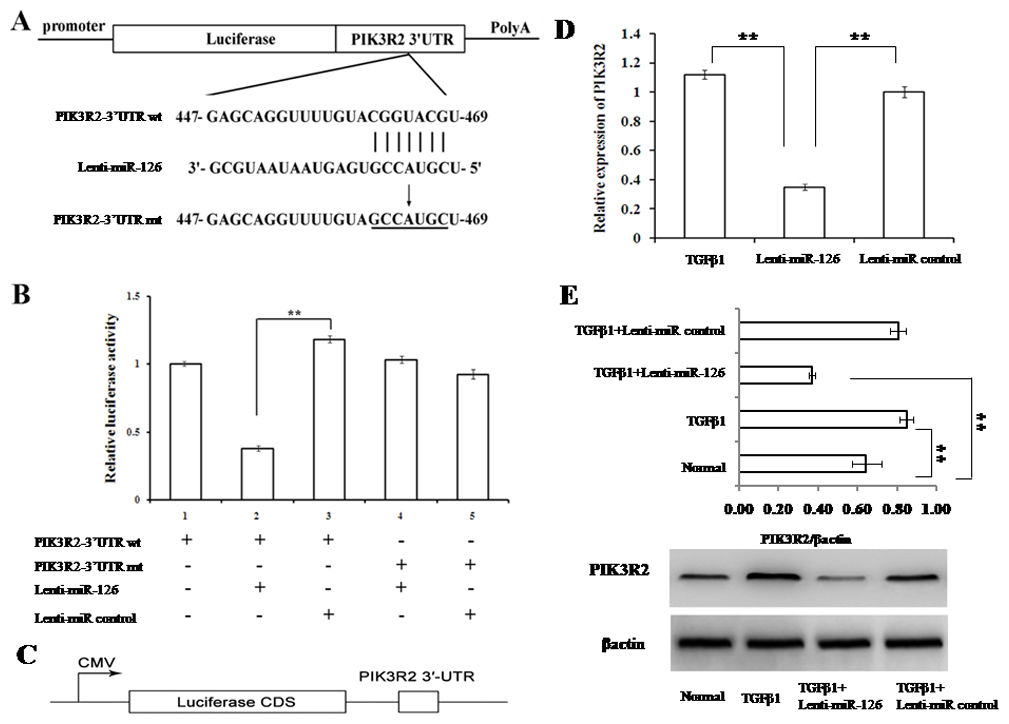
PIK3R2 was a direct target of miR-126 in EPCs. (A) Diagram of *PIK3R2*-3′UTR-containing luciferase reporter gene construct and the 22-bp target site of miR-126 in *PIK3R2*-3′UTR. The mutated nucleotides in *PIK3R2*-3′UTR fragments are underlined. (B) Luciferase reporter assays. The wild-type or mutant reporter plasmids were cotransfected into EPCs, which were infected by control-lentivirus or miR-126-lentivirus. The relative luciferase values shown were normalized to transfections with the wild-type reporter plasmids. Values are the average ± S.D. of 3 replicates. (C)The scheme of the luciferase assay to evaluate the direct inhibition of mir-126 on PIK3R2 protein expression. (D) The expression levels of PIK3R2 mRNA in miR-126 EPCs, miR-126 control, and the negative control—all under TGFβ1 treatment—were examined by qRT-PCR. β-actin was used as an internal control. (E) The protein expression of PIK3R2 was examined by western blotting. β-actin was used as an internal control. ** represents *P* < 0.01 with an LSD t-test.

After an extensive review of online microRNA database by using TargetScan, picTar and RNA22, we selected PIK3R2 as a putative target gene of miR-126 in EPCs. To confirm that *PIK3R2* is a direct target of miR-126 in EPCs, we cloned a 22-bp fragment of the target sequence of the *PIK3R2* 3′UTR (wt 3′UTR) or the mutant sequence (mt 3′UTR) into a luciferase reporter vector, downstream of the reporter gene (Figure 3A). EPCs or miR-126 overexpressing EPCs were then transfected with the wt or mt 3′UTR vector. The results showed a significant decrease in luciferase activity in EPCs when compared with the miR control, while no significant difference in luciferase activity was detected when the miR-126 seed-binding sites of PIK3R2 were mutated (Figure 3B). In addition, we determined the impact of miR-126 on PIK3R2 in EPCs and TGFβ1-treated EPCs since both mRNA and protein expression levels of PIK3R2 significantly decreased (Figure 3C & 3D). Compared to the levels for the normal control, the PIK3R2 protein level was elevated markedly by TGFβ1 (Figure 3D). These results indicate that *PIK3R2* is involved in the EndMT mechanism of miR-126 in EPCs.

**Figure 4:**
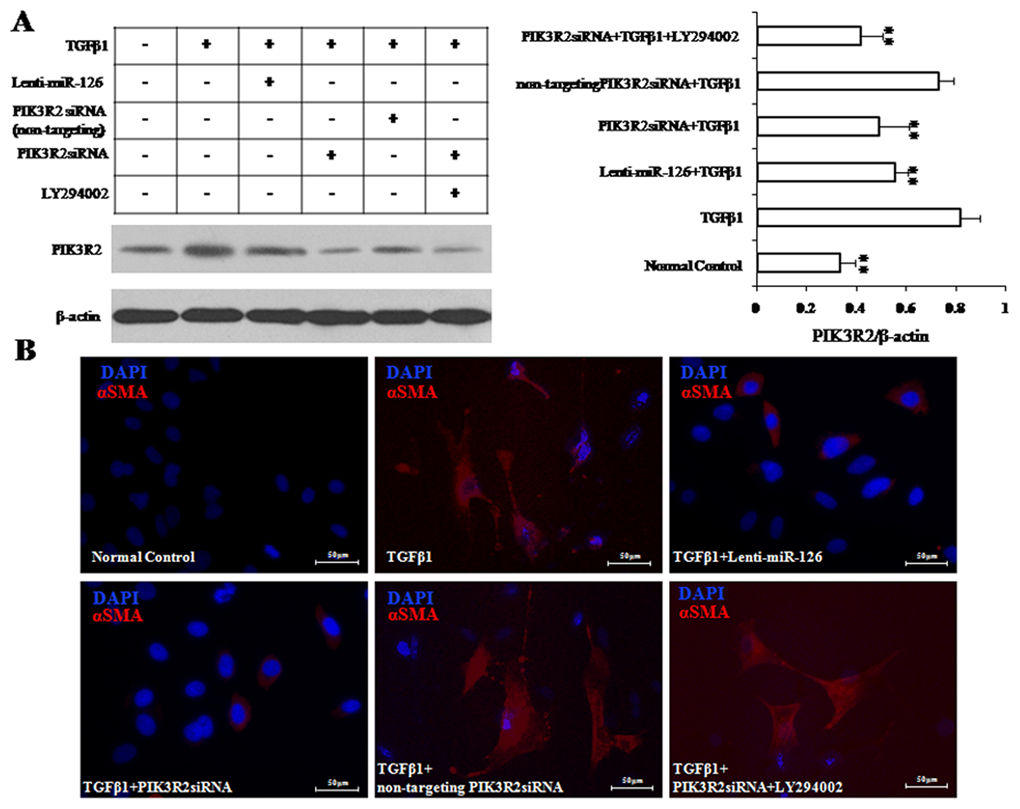
miR-126 inhibits EPC EndMT via targeting PIK3R2. (A) PIK3R2 expression was down-regulated by PIK3R2siRNA. (B) After the pre-treatment with PIK3R2siRNA, EPCs were treated TGFβ1 (5 ng/mL) for 7 days to induce EndMT. The αSMA expressions were detected by using immunofluorescence staining (Bar=50 μM; Blue for DAPI; Red for αSMA). ** represents *P* < 0.01 with an LSD t-test.

To demonstrate that PIK3R2 is the target gene of miR-126 during EndMT process in EPCs, we performed the loss-of-function experiment by knocking down PIK3R2 gene expression level to mimic the same effect of miR-126 on EPCs (Figure 4). In details, we transfected EPCs with lenti-miR-126 in the presence or absence of LY294002, a potential inhibitor of PIK3. Then we co-transfected EPCs with the specific siRNA against the rat PIK3R2 gene (PIK3R2 siRNA). The effect of siRNA against PIK3R2 genes was validated by Western blot analysis (Figure 4A). After treatment with TGFβ1 (5 ng/mL) for 7 days, we examined morphological changes as well as gene expression profile as described in Figure 1 and 2. We found that knockdown of PIK3R2 significantly reversed the morphological changes of EPCs from cobblestone-like to spindle-shape appearance in the presence of TGFβ1, similar to the findings observed in the lenti-miR-126 treated EPCs (Figure 4B). In addition, the characteristic decreased α-SMA gene expression profiles followed the similar pattern of changes between lenti-miR-126 treated and knockdown of PIK3R2 group in EPCs (Figure 4B). LY294002 is a specific, potent PIK3 inhibitor that blocks the PIK3/Akt signal transduction pathway. Interestingly, we have shown that LY294002 could completely reversed the inhibitory effect on EndMT by PIK3R2 knockdown on TGFβ1 treated EPC (Figure 4B). In other words, in th presence of PIK3R2 knockdown, LY294002 restored the EndMT characteristic morphological changes to spindle-shape appearance as well as expression marker changes with increased α-SMA level in those cells. These results suggest that miR-126 regulates the EndMT process via the direct target gene of PIK3R2, and involves the PI3K-Akt signaling pathways.

### miR-126 regulates PI3K/Akt signalling pathways involved in TGFβ1-induced EndMT of EPCs

**Figure 5:**
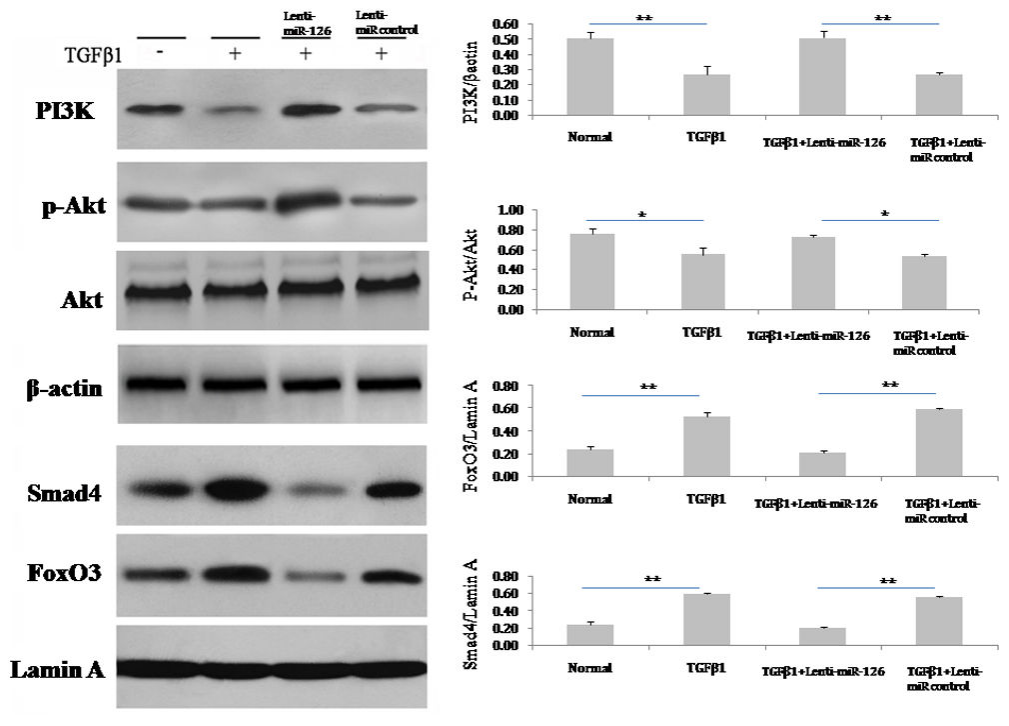
miR-126 activated the PI3K/Akt and inhibited the FoxO3/Smad4 signalling pathways in TGFβ1-induced EPCs. After the treatment with TGFβ1 (5 ng/mL) for 7 days, the protein in EPCs were prepared. Immunoblotting assays were performed using specific antibodies against PI3K, phosphor-Akt, Akt, FoxO3, and Smad4. β-actin and lamin A were used as internal controls for total cells and the nuclear proteins assay separately. The relative protein levels of these proteins were determined by densitometry analysis (n = 3). Data are shown as mean ± S.D. values. * and ** represent P < 0.05 and P < 0.01 with an LSD t-test, respectively. The number of observations (n) represents the number of independent cell preparations.

Normal EPCs expressed the PI3K and phosphor-Akt proteins at a basal level. When EPCs were treated with TGFβ1, the protein levels of PI3K and phosphor-Akt decreased noticeably, which were partially reversed to basal levels by overexpression of miR-126 (Figure 5) and not by the miR-126 empty vector. TGFβ1 did not cause a change in the total Akt protein level. Additionally, MiR-126 did not affect the total Akt protein level, regardless of TGFβ1 treatment. 

### miR-126 regulates Smad4/FoxO3 signalling pathways involved in TGFβ1-induced EndMT of EPCs

The nuclear proteins FoxO3 and Smad4 were expressed in EPCs at a basal level, and the levels were elevated markedly by TGFβ1 treatment. MiR-126, but not the miR-126 empty vector decreased the protein levels of FoxO3 and Smad4 in TGF-β1-treated EPCs (Figure 5).

## Discussion

EPCs are considered potential candidates for coronary heart disease treatment because they can differentiate into mature vascular endothelial cells. However, there is accumulating evidence that EPCs are also capable of differentiating into mesenchymal cells to aggravate intimal hyperplasia that contributes to atherogenesis [4,6]. *In vitro*, EPCs could undergo the EndMT process upon treatment with TGFβ1 [2]. *In vivo*, EPCs transplanted to impaired endothelium enhanced intimal hyperplasia, but only half of the EPCs underwent a transition to smooth muscle cells (SMCs) induced by TGFβ1 [5]. These results call into question whether the EndMT process of EPCs could be regulated. Here, our experiments show that deficiency of miR-126 expression is, at least in part, responsible for the EndMT of EPCs induced by TGFβ1. In addition, we found that miR-126 interacts with its target gene *PIK3R2* to inhibit the EndMT process, involving regulation of the PI3K/Akt-FoxO3/Smad4 signalling pathway.

Mature endothelial cells can also transdifferentiate to SMC-like cells by the EndMT process [11]. In our preliminary experiments, EPCs under stimulation with TGFβ1 displayed protein expression of αSMA in 24 hours, while the endothelial cell marker *CD31* decreased markedly ( Figure S2). Our results clearly showed that the EndMT observed in this study occurred as differentiation from EPCs and that EndMT did not occur indirectly from mature endothelial cells. A previous study [5] showed that PCs derived from rat bone marrow were able to undergo EndMT with TGFβ1 treatment alone; however, human circulating EPCs can only be differentiated into mesenchymal cells under cell-to-cell contact with smooth muscle cells [6]. Taken together, these results demonstrate the more complex issues of human EndMT regulation.

miRNAs have been widely reported to be involved in transdifferentiation of progenitor and stem cells [23]. miR-126, an endothelial-specific miRNA enriched in EPCs, regulates EPC proliferation, mobilization, and migration [9,14]. In the present study, miR-126 levels decreased significantly in the EPC EndMT induced by TGFβ1, suggesting that miR-126 could be involved in the EndMT process of EPCs. To further confirm the role of miR-126 in EPC EndMT, overexpression of miR-126 was achieved in EPCs by transfection with a lentivirus vector encoding miR-126. As a result of miR-126 overexpression in EPCs undergoing the EndMT process, we observed the diminished mRNA expression of mesenchymal cell markers, such as *αSMA*, *sm22*, and myocardin. Cytoplasmic expression of *αSMA* in EPCs treated with TGFβ1 was inhibited markedly by miR-126. These results demonstrated that miR-126 inhibited the EndMT process of EPCs.

Epidermal growth factor-like domain 7, Egfl7, is a largely endothelial restricted gene which is thought to have a role during the differentiation of embryonic stem cells (ESCs) along the endothelial lineage [12]. After treatment of EPCs with TGFβ1 (5 ng/mL) for 7 days, we found that the mRNA expression level of Egfl7 did not alter while miR-126 expression level decreased gradually along the 7 day treatment course ( Figure S3). However, in the lentiviral miR-126 treated EPCs, we did not observe any mRNA expression level changes of Egfl17 ( Figure S3A). Based on those findings, we conclude that Egfl7 is not involved in the EndMT process of EPCs. The mechanism of miR-126 downregulation by TGFβ1 is prabobaly through the Egfl7 T-2 promoter containing CpG islands. TGFβ1 may induce hypermethylation in T-2 promoter which in turn silences intronic miR-126 located within the 7th intron of the host Eglf7 gene. The physiological significance of miR-126 and Egfl7 is distinct as demonstrated knockout mice model[24]. They used selectively floxed Egfl7 (Delta) and miR-126 (Delta) alleles to reveal that Egfl7 (Delta/Delta) mice were phenotypically normal, whereas miR-126 (Delta/Delta) mice bearing a 289-nt microdeletion recapitulated previously described Egfl7 embryonic and postnatal retinal vascular phenotypes.

To explore the mechanisms of miR-126 in the regulation of EPC EndMT, we confirmed that the gene coding for PI3K regulatory subunit p85 beta (*PIK3R2*), a target gene of miR-126 identified previously in endothelial cells [16], is also the target gene of miR-126 in EPCs by using a luciferase reporter assay, consistent with the findings in circulating EPCs in preeclampsia[25]. Additionally, we found that PIK3R2 protein levels were increased markedly in TGFβ1-treated EPCs and that levels were significantly decreased by miR-126, suggesting that PIK3R2 was involved in the negative regulation of miR-126 in EPC EndMT. Next, we investigated if PIK3R2 is the target gene regulated by miR-126 to inhibit the EndMT process in EPCs. We used siRNA to knockdown PIK3R2 expression level in EPCs. Consistent with overexpression of miR-126 in EPCs, we found that PIK3R2 knockdown had similar effect to block TGFβ1 induced EndMT process. This conclude that miR126 inhibits EndMT in EPCs by down-regulating the direct target gene of PIK3R2.

Phosphatidylinositol 3-kinase (PI3K) is a lipid kinase that phosphorylates phosphatidylinositol and similar compounds, creating second messengers important in growth signaling pathways. PI3K functions as a heterodimer of a regulatory unit p85 and a catalytic subunit p110. P85 units binds to activated (phosphorylated) protein-tyrosine kinases, through its SH2 domain, and acts as an adapter, mediating the association of the p110 catalytic unit to the plasma membrane. PIK3R2 is one of the PI3K p85 subunit family members. Contrary to PIK3R1, PIK3R2 acts as a suppressor of the PI3K/Akt signalling pathway activation, thereby inhibiting the pathway [26]. In the present study, phosphor-Akt levels decreased markedly in TGFβ1-treated EPCs, whereas levels increased notably by overexpression of miR-126. These results are consistent with the previous findings in mesenchymal stem cells that miR-126 induced phosphorylation of Akt without exogenous stimulus [27]. Furthermore, recent studies have shown that the inhibition of PI3K/Akt activation by wortmannin, an inhibitor of PI3-kinase, increases αSMA expression in EPCs under shear stress [28], indicating a inhibitory role of PI3K/Akt activation in EndMT of EPCs. Taken together, these findings imply that miR-126 inhibited EPC EndMT via targeting *PIk3R2* to enhance PI3K/Akt activation. Interestingly in our study, blocking PI3K/Akt activation by PI3K inhibitor LY294002 could completely reverse the inhibitory effect of knockdown PI3KR2 on αSMA expression as well as the morphological changes of EPCs in EndMT process. Taken together, our findings imply that miR-126 decreases PIk3R2 expression to enhance PI3K/Akt activation, thereby inhibiting EndMT in EPCs.

TGFβ1 is an important inducer for EPC EndMT, in addition to platelet-derived growth factor-BB [29]. Specifically, the TGFβ receptor I (TGFβR I)-dependent-Smad2/3/4 signalling pathway is responsible for TGFβ1-induced EPC EndMT [6]. FoxO3, a member of the Forkhead box (Fox) family, has been found to be a key partner for Smad3 and Smad4, which were activated by TGFβ1 [20]. Upon activation, Smad3 and Smad4 (not Smad2) formed a complex with FoxO3, localized to the nucleus, and coupled with different transcription factors [30], thereby playing an essential role in cell differentiation [31]. In this study, miR-126 inhibited EPC EndMT, accompanied by a significant decrease in nuclear FoxO3 and Smad4 levels, suggesting a role of the FoxO3/Smad4 complex in the negative regulation of miR-126 in EPC EndMT. Interestingly, the FoxO3/Smad4 complex formation is negatively regulated by PI3K/Akt activation, which leads to localization of FoxO3 from the nucleus to the cytoplasm [32]. Augmentation of Akt phosphorylation has been found to inactivate the FoxO3 pathway in EPCs [33]. Interestingly, Akt inhibits Smads, not by phosphorylation, but by direct interaction with Smads proteins and sequesters the Smads complex to the cytoplasm, thereby inhibiting its transcriptional capability (26). Given our current results that overexpression of miR-126 enhanced the level of phosphor-Akt, our findings indicate that miR-126 might promote PI3K/Akt activation to negatively regulate the FoxO3/Smad4 signalling pathway. The exact mechanism underlying the pathway inhibiting EPC EndMT needs further examination. Moreover, the current results in our study did not directly prove that the FoxO3/Smad4 complex or their respective isoforms were indispensable in EPC EndMT, which remains to be elucidated. 

In addition, previous studies showed that EPCs undergoing EndMT would synthesize and secrete TGFβ1, which might induce EndMT in other EPCs in a paracrine mechanism [5]. It remains unclear whether miR-126 inhibition of the EndMT process is indirectly controlled by decreasing the secretion of TGFβ1 from EPCs undergoing EndMT.

Notably, in our study, miR-126 was found to inhibit the decrease in mRNA levels of the progenitor cell markers, *CD133* and *CD34*, implying an important role for miR-126 in maintaining the EPCs progenitor properties. Previous studies have demonstrated that the PI3K/Akt/FoxO3 signalling pathway is critical for the self-renewal of hematopoietic stem cells [34], which are derived from bone marrow, similar to EPCs used in this study. In addition, it will be interesting to explore whether the PI3K/Akt/FoxO3 signalling pathway, under the regulation of miR-126, would be involved in homeostasis of EPCs. 

Additionally, previous studies have demonstrated that the mesenchymal genes in circulating EPCs could not be upregulated by the 48-hour treatment with TGFβ1 [6]; however, in this study, the mesenchymal genes in BM-derived EPCs were markedly elevated by 7-day treatment with TGFβ1. The inconsistency might be due to the different times of TGFβ1 stimulus, and it might also result from the disparity in basal levels of miR-126 between circulating EPCs and BM-derived EPCs, which should be explored further. 

One of the limitations of our study is the selection of PIK3R2 as a putative target gene based on review of online microRNA database using bioinformatics tools. However, we do understand that the only way to reveal all other possible target genes is to study the mRNA microarray data from EPCs infected with lenti-miR-126. Therefore, future research will be needed to investigate other interesting target genes or pathways regulated by miR-126 in EPCs.

In summary, we found that overexpression of miR-126 inhibited EndMT of EPCs. This inhibition likely occurred by interaction with the target gene *PI3KR2* to regulate the PI3K/Akt signalling pathway, thus regulating gene expression through the FoxO3/Smad4 transcription complexes. Our research provides a tool to deliver vector-based miR-126 to an endothelial system, thereby inhibiting intimal hyperplasia through the EndMT mechanism. This vector-based tool could be a novel therapeutic treatment for in-stent restenosis of coronary artery disease, a common problem of interventional cardiology practice. Furthermore, overexpression of miR-126 could be a potential therapy for pulmonary vascular intima hyperplasia in pulmonary hypertension, a challenging disease in medicine. Future research should be focused on investigating the diagnostic and therapeutic values of miR-126 in those cardiovascular diseases. 

## Supporting Information

Figure S1 **MiR-126-lentivirus transfection efficiency.** EPCs were transfected with lenti-miR-126 and lenti-GFP (MOI = 10) for 48 hours, and the transfection efficiency was detected by immunofluorescence (A). By using flow cytometry analysis, the transfection efficiency of lenti-miR-126 and lenti-GFP reached 87.56% and 90.91% respectively (B). Endogenous miR-126 expression in lenti-miR-126-transfected EPCs increased to 3.6 times compared with the vehicle control by using quantitative real time-PCR (C). U6 was used as an internal control. Data are shown as mean ± S.D. (n = 3).**, *P* < 0.01 compared with EPCs infected with lenti-miR-126. The number of observations (n) represents the number of independent cell preparations.
(TIF)

Figure S2 **Chronological change in protein expression of α-SMA and CD31 in the process of EndMT.** EPCs were treated with or without TGFβ1 (5 ng/mL) for 1, 4, and 7 days. The protein expression of α-SMA and CD31 was examined at each time point by using western blot assay.
(TIF)

Figure S3 **miR-126 on Egfl7 expression in EPC EndMT.** EPCs were treated with TGFβ1 (5 ng/mL) for 1, 3, 5 and 7 days, and miR-126 relative expression was detected by using quantitative real time-PCR (qRT-PCR).U6 was used as internal control. (B) After 7-day treatment with TGFβ1 (5 ng/mL), Egfl7mRNA expressions were detected in all groups by using qRT-PCR. Β-actin was used as an internal control. EPCs without any treatment were used as the normal control.**, *P* < 0.01 compared with EPCs without any treatment.
(TIF)
