# Supplementary figures and images for: microRNA 126 Inhibits the Transition of Endothelial Progenitor Cells to Mesenchymal Cells via the PIK3R2-PI3K/Akt Signalling Pathway
Source: PLoS One. 2013 Dec 13;8(12):e83294. doi: 10.1371/journal.pone.0083294 (PMC3862723; doi:10.1371/journal.pone.0083294)

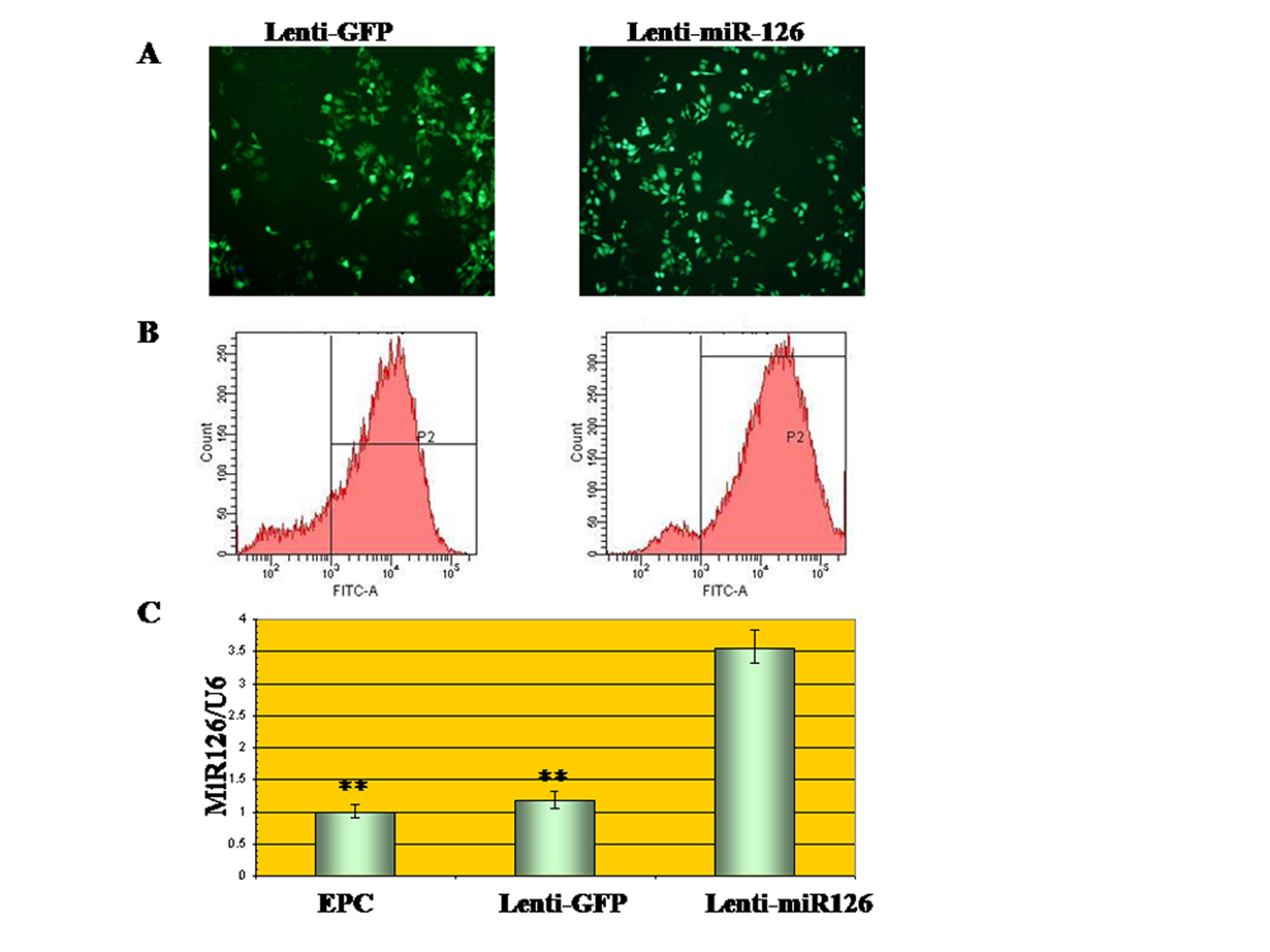

Supplement: Figure S1 — MiR-126-lentivirus transfection efficiency. EPCs were transfected with lenti-miR-126 and lenti-GFP (MOI = 10) for 48 hours, and the transfection efficiency was detected by immunofluorescence (A). By using flow cytometry analysis, the transfection efficiency of lenti-miR-126 and lenti-GFP reached 87.56% and 90.91% respectively (B). Endogenous miR-126 expression in lenti-miR-126-transfected EPCs increased to 3.6 times compared with the vehicle control by using quantitative real time-PCR (C). U6 was used as an internal control. Data are shown as mean ± S.D. (n = 3).**, P < 0.01 compared with EPCs infected with lenti-miR-126. The number of observations (n) represents the number of independent cell preparations. (TIF) [file pone.0083294.s001.tif]

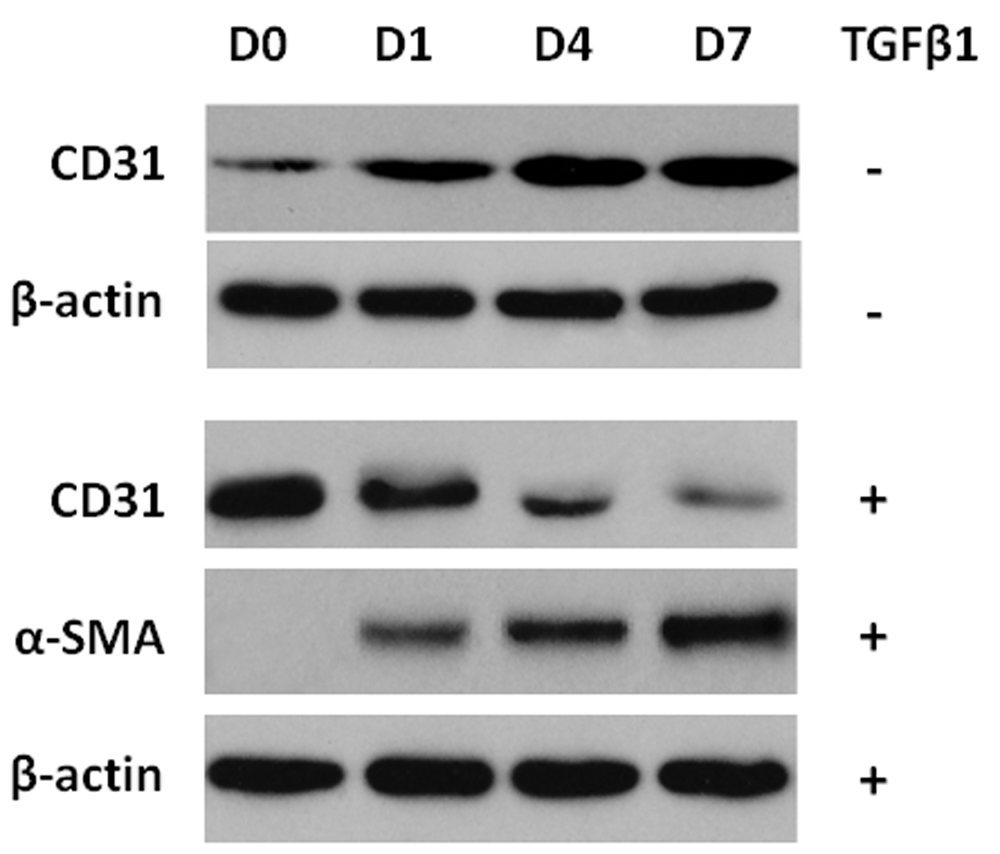

Supplement: Figure S2 — Chronological change in protein expression of α-SMA and CD31 in the process of EndMT. EPCs were treated with or without TGFβ1 (5 ng/mL) for 1, 4, and 7 days. The protein expression of α-SMA and CD31 was examined at each time point by using western blot assay. (TIF) [file pone.0083294.s002.tif]

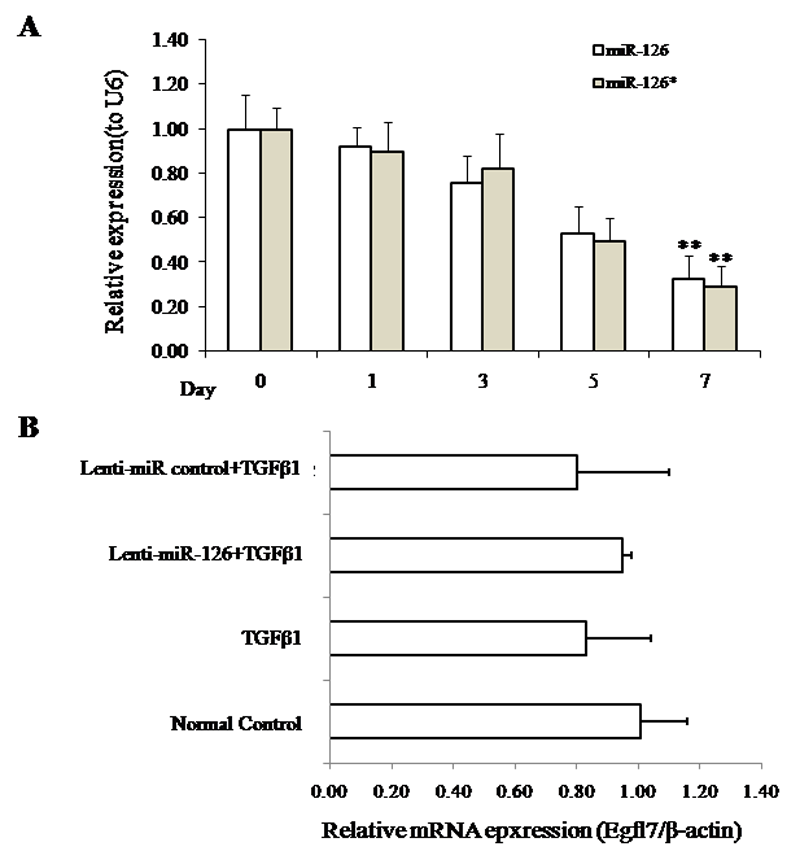

Supplement: Figure S3 — miR-126 on Egfl7 expression in EPC EndMT. EPCs were treated with TGFβ1 (5 ng/mL) for 1, 3, 5 and 7 days, and miR-126 relative expression was detected by using quantitative real time-PCR (qRT-PCR).U6 was used as internal control. (B) After 7-day treatment with TGFβ1 (5 ng/mL), Egfl7mRNA expressions were detected in all groups by using qRT-PCR. Β-actin was used as an internal control. EPCs without any treatment were used as the normal control.**, P < 0.01 compared with EPCs without any treatment. (TIF) [file pone.0083294.s003.tif]
